# The Political Economy of Digital Health Equity: Structural Analysis

**DOI:** 10.2196/46971

**Published:** 2024-03-26

**Authors:** James Shaw, Wiljeana Glover

**Affiliations:** 1 Department of Physical Therapy Temerty Faculty of Medicine University of Toronto Toronto, ON Canada; 2 Joint Centre for Bioethics, Dalla Lana School of Public Health, University of Toronto Toronto, ON Canada; 3 Technology, Operations, and Information Management Division Babson College Wellesley, MA United States

**Keywords:** digital health equity, health equity, health policy, telemedicine, digital care, political economy, race, ethnicity, socioeconomic, policy

## Abstract

Digital technologies have produced many innovations in care delivery and enabled continuity of care for many people when in-person care was impossible. However, a growing body of research suggests that digital health can also exacerbate health inequities for those excluded from its benefits for reasons of cost, digital literacy, and structural discrimination related to characteristics such as age, race, ethnicity, and socioeconomic status. In this paper, we draw on a political economy perspective to examine structural barriers to progress in advancing digital health equity at the policy level. Considering the incentive structures and investments of powerful actors in the field, we outline how characteristics of neoliberal capitalism in Western contexts produce and sustain digital health inequities by describing 6 structural challenges to the effort to promote health equity through digital health, as follows: (1) the revenue-first incentives of technology corporations, (2) the influence of venture capital, (3) inequitable access to the internet and digital devices, (4) underinvestment in digital health literacy, (5) uncertainty about future reimbursement of digital health, and (6) justified mistrust of digital health. Building on these important challenges, we propose future immediate and long-term directions for work to support meaningful change for digital health equity.

## Introduction

Digital health has become an integral component of health care delivery [1-3]. Although the use of digital technologies to deliver care has been significantly accelerated by the COVID-19 pandemic [3], the trend toward digitally enabled health care has been progressing for several years alongside the growing power of the technology industry and the widespread distribution of digital devices and infrastructure [4-6]. Digital technologies have produced many innovations in care delivery and enabled continuity of care for many populations when in-person care was challenging or impossible. However, a growing body of research suggests that digital health can also exacerbate health inequities for those excluded from its benefits for reasons of cost, digital literacy, and structural discrimination based on age, race, ethnicity, or socioeconomic status [7,8].

Often referred to as the “health-related digital divide,” the exacerbation of health inequities by digital health technologies has been widely discussed in health informatics and digital health literature [7,9,10]. Now that the digital divide has widened as a result of the COVID-19 pandemic, strategies to address its impacts on health have been more widely discussed at policy, health system leadership, clinical, and community levels [8,11]. Building on past research bringing critical perspectives to bear on health equity and digital innovation [12], we argue that these recommended practices and policies have not yet made meaningful progress on digital health equity in part because scholars, providers, policy makers, and advocates have not yet adequately confronted the social and structural influences that reinforce the status quo at the digital health and health care industry nexus.

In our paper, we draw on a political economy perspective that brings attention to the incentives and actions of powerful stakeholders in a given field [13,14], in this case primarily government, professional, and corporate actors who hold the power to shape the digital health landscape. Considering the incentive structures and investments of such powerful actors, we outline how characteristics of neoliberal capitalism in Western contexts produce and sustain digital health inequities by describing 6 structural challenges to the effort to promote health equity through digital health. Acknowledging that the concept of “digital health equity” has been defined in different ways [15,16], we focus here on digital health equity as an aspirational state in which all communities have access to digital health technologies that enable them to meet their health-related needs. Such a state can be defined at different scales, for example, within a patient population, a city, a province or state, an entire country, or worldwide. Achieving the goal of digital health equity requires commitments from diverse actors in various settings, which we explore in detail in our paper.

Instead of reproducing frameworks proposing multiple points of action to redress digital health inequities [10,15,17], in the concluding sections of our paper, we highlight what we understand to be a central tension inherent in efforts to enhance digital health equity. Specifically, we discuss the challenge of supporting commercial actors to invest in immediate actions that enhance digital health equity while acknowledging that the broader health care systems and business models through which they operate can be obstacles to the meaningful advancement of equity and justice for people’s health. We conclude by outlining the need for immediate and long-term directions for research, policy, and advocacy for digital health equity, one focused on foundational reform and the other on stronger collaboration to deepen the commitments of commercial organizations to equity in digital health.

## Defining Digital Health

In its broadest sense, digital health refers to a diverse collection of technologies and approaches to care delivery that uses digital technologies or the data they generate to inform health care, self-management, and public health [4,18,19]. Digital health can be understood to include at least 4 distinct groups of applications of digital technology to achieve health-related aims, as outlined in Textbox 1 [20,21]. This broad sense of digital health introduces the wide variety of use cases that are considered when policy makers contemplate digital health strategies as solutions to problems in health care and public health.

**Definition of digital health.**

Digital health refers to a diverse collection of technologies and their uses to achieve health-related goals. We identify the following 4 groups of digital health use cases [20]:

1. The collection of data about health and health care into electronic patient records to inform service planning and care delivery
2. Digital apps (usually used on smartphones) that support people to engage with their health and self-monitoring in an ongoing way
3. Telemedicine and internet-based care strategies that enable health care to be delivered at a distance
4. Analytics of health-related data from multiple sources, including Internet of Things devices, to generate new health-related insights and applications.

The hopes for what digital health can accomplish are high. One highly cited commentary on evaluation standards for digital health stated that “digital health interventions have enormous potential as scalable tools to improve health and health care delivery by improving effectiveness, efficiency, accessibility, safety, and personalization” [5]. The variety of goals embedded in this statement conveys a general sense that digital health strategies can help to achieve many of the most elusive goals of health care and public health. For example, the notion that digital health apps can lead patients to be more deeply engaged in their own health and well-being, becoming more active participants in their own wellness and care [22,23], or that electronic patient records can enhance collaboration between providers to produce more integrated health care experiences for patients [24]. If successfully implemented, these specific applications of digital health technologies would contribute to improving health care in profound ways.

## Digital Health and Health Inequities

Despite these high ambitions, the positive impacts of digital health have not been evenly distributed. Over the past several years, researchers have documented the exclusion of particular communities from the benefits of digital health technologies in both high-income and low-income country settings [8,25-27]. Systematic reviews of this literature have consistently illustrated that communities excluded from the benefits of digital health are those communities facing forms of structural oppression, including economic exclusion, racism, housing injustice, ableism, ageism, and others [9,28-30]. For example, multiple systematic reviews have reported the exclusions of Indigenous peoples in North America and Australia from engaging with digital health technologies as a result of a lack of cultural safety in applications of digital health [17,31]. Communities speaking a first language other than English have also been excluded from the benefits of digital health, for example, in situations that demand people navigate a complex digital interface for mental health support [32]. Communities with lower levels of digital literacy or digital readiness as a result of a lack of educational opportunities also face added barriers to engaging in digital health; for example, older people with less exposure to digital technology and those living with lower incomes who lack the financial resources to afford such technologies in the first place [8,17,25]. The reliance on digital health during the COVID-19 pandemic has only exacerbated these realities, leading to deeper inequities in access to health care that stand to have important longer-term consequences [33-36]. For example, the accelerated adoption of internet-based care and other digital health technologies has led to a range of inequities in access to care [36], and although not enough time has passed to know the long-term impacts of these technologies, they are very likely to be a permanent element of care delivery.

A number of authors have proposed frameworks for understanding where, in the design, adoption, and use of digital health technologies, these exclusions occur in order to identify likely points of redress [9,17,26]. Such works have emphasized the importance of a multilevel approach to addressing digital health inequities, generally proposing interventions at the levels of policy, health system leadership, clinical practice, and patient or community engagement [9,11,16]. These strategies, some of which are summarized in Table 1, constitute identifiable actions for specific stakeholders to engage in the effort to enhance health equity in the field of digital health. However, beyond a few notable examples [37], there is little indication that meaningful progress has been made in adopting these strategies across digital health stakeholders. In this paper, we explore why that is the case by pointing out structural obstacles to advancing digital health equity in the broader digital health ecosystem.

**Table 1:** Sample strategies to promote digital health equity (adapted from Shaw et al [9]).

Stakeholder group responsible and domain of intervention		Example strategies
**Policy and government**		
	Government policy	Government policy should clarify standards for the inclusive design of digital health innovations.
	Funder (reimbursement)	Ensure payment parity between insurers for video and audio visits.
	Access to devices	Promote access to broadband internet, especially among those who cannot afford it.
	Health-related messaging	Increase emphasis on and diffusion of culturally relevant public health messages (eg, increase redundancy of important messaging).
**Organization and health system**		
	Health care organizations	Measurement of equity-related outcomes, such as the number of visits using interpreter services.
	Clinical practice	Provide training and support to patients seeking to access care on the internet.
**Community and patient**		
	Community engagement	Partner with community organizations to provide peer-led educational support.
	Enhancing digital literacy	Mitigate digital literacy and resource barriers (eg, provide patient education to enhance digital literacy skills and inform patients about free or reduced-cost internet access locations).

## Political Economic Analysis: A Structural Approach

We approach our analysis from a political economy perspective on digital health inequities. The concept of political economy has been used in many ways in relation to health care and public health [38-40]. For our purposes, we describe political economy as a set of relationships and incentives that characterize the interconnections between state, corporate, and other actors with respect to a given domain of social activity, in our case, digital health. Central to analyses of political economies is the concept of power, understood in this context as the capacity of certain actors to exert influence over states of affairs in order to produce outcomes favorable to their interests [27,39]. Power is a complex and productive phenomenon embedded in systems of policy institutions, economic activity, and globalization that make such influence possible [41].

Analyses of political economy build on a clear understanding of the links between systems of policy or public administration and the economic regimes in place in a given jurisdiction. In North American and European contexts, the impacts of neoliberal capitalism on population health have garnered substantial attention [31,42,43]. In this sense, a core feature of neoliberal capitalism is the favoring of private enterprise and the reduction in capacity and responsibility of government or public services [42]. For example, Friel et al [41] showed how neoliberal policy approaches to health-related reforms in Australia led to the favoring of private enterprise and individualized strategies as opposed to structural- or population-level initiatives intended to improve health.

With respect to digital health, the policy framework and incentive structures of neoliberalism incentivize the development of products and technologies with a maximum capacity for scale because maximal scale generates maximal profits [42,43]. While neoliberalism has apparently led to a population-level decrease in poverty through economic growth, it has also increased financial and power inequities that have important impacts on health [44]. Given their capacity to explain whether and how large-scale change occurs in political and economic regimes, political economy approaches have been particularly useful in understanding why more profound action is not taken to address the social determinants of health by states, health care funders, and health systems [41,45].

In these ways, a political economy approach to digital health in the context of neoliberalism examines the influence held by powerful actors over investment and innovation in digital health and the capacity of the state or other actors to steer these processes in the public’s interest. Reflecting on the political economy of digital health, Storeng et al [27] exclaimed that “new actors, financial instruments, (absent) legal frameworks, and the power structures of capitalism drive the innovation, development, and applications of digital technologies, reshaping health systems, health care, and medicine.” We make observations in our analysis informed by these remarks about political economy, describing the impact of active investments by some actors and inaction by others on the possibilities of achieving digital health equity at scale.

In our analysis, we sought to take an innovation ecosystem perspective [46], beginning with a broader set of influences on digital health inequities and concluding with a narrower focus on health care delivery and patients. Specifically, we aimed to document the impacts of powerful political and economic actors that shape the broader ecosystem in which digital health innovation takes place. These actors include large corporations, venture capitalists, digital health innovators, and policy makers who set the terms of engagement in digital health. Although policy and investments in digital health are a central consideration, we also acknowledged that the influence of broader policy related to digital inclusion is essential to consider (eg, decisions regarding where high-speed internet is made available and how education regarding digital technologies will be provided). We then narrowed our perspective to focus on health care delivery systems and the patients with whom they engage, documenting decision-making and circumstances that impact digital health inequities at the interface between provider organizations and the communities they serve. Our observations were directly informed by the political economy perspective outlined here.

## Structural Barriers to Digital Health Equity

### The Role of Large Technology Corporations

The first challenge we outline relates to the role of large technology corporations in the digital health innovation ecosystem. Large technology companies have come to enjoy immense influence over our everyday lives, and this influence extends deeply into health care [46]. The COVID-19 pandemic witnessed large technology corporations taking advantage of the need for rapid responses to issues such as contact tracing, with the Apple-Google contact tracing application programming interface becoming central to several countries’ contact tracing efforts [14,47]. However, investments from technology corporations extend well beyond contact tracing. Meta (Facebook), Apple, Microsoft, Google, and Amazon invested a combined US $6.8 billion in health care between January 2020 and June 2021 [48]. Investments ranged from Microsoft’s purchase of a health care chatbot company to Facebook’s development of a preventative health tool to promote regular health screening [48]. With the immense resources held by large technology corporations and their persistent investment in acquiring market share for digital technologies in health care, they stand to gain substantial influence over the structure and delivery of health care [49].

The influence of large technology companies may limit the progress that can be made in advancing digital health equity. The incentives of large corporations are fundamentally driven toward the scaling of technology offerings and the profit they generate [6]. Innovations developed in large corporate environments are those that can be marketed to a segment of the population (or some other payer) that is capable of paying market prices [8,14]. The disposable income or connection to other forms of financial capital (eg, generous health insurance plans) required to access these innovations as they arrive on the market excludes communities that are economically marginalized. This means that the incentives characterizing the activity of technology corporations are fundamentally different from those for achieving equitable access to and outcomes of digital health. Even applications that are free to end users, for example, contact-tracing apps, raise issues of privacy, security, access, and equity for cultural minority and other structurally marginalized patient populations because app rollout may not target these populations [50]. By supporting and incentivizing technologies that work well for the economically privileged and creating challenges for other actors aiming to provide free or low-cost access to technologies, the actions and incentives of large technology corporations risk widening the divide between those who benefit from access to digital health and those who do not.

### Innovation Policy and the Venture Capital System

Beyond the power and impact of large technology corporations, smaller-scale digital health startups are constantly appearing in the digital health ecosystem [51]. These companies are established and developed through a variety of routes and seek out financial support to fund their activities progressing toward implementation and scale [52]. In some cases, health systems actively invest in digital health startups as a strategy to promote innovation that more directly addresses clinical and operational needs [53]. In these cases, an explicit focus on digital innovations that promote health equity is far more likely [16]. However, external venture capital investment in digital health startups is responsible for the largest portion of venture capital funding. Despite recent declines in new investments due to a market downturn, digital health startups worldwide attracted a combined US $30.7 billion of venture capital investments in 2021, with telemedicine, data analytics, and health-related mobile apps being the highest investment categories [54].

Importantly, venture capital investors have a deep influence over the development and direction of technologies and business models in health-focused start-ups [55,56]. In a series of papers reporting research on the role of venture capital in health care technology companies, Lehoux and colleagues [52,55,56] illustrated the ways that Canadian venture capital investors actively shape the activities of startups and influence the value proposition of the technology being developed. Investors retain a primary incentive to maximize future potential earnings and contribute to shaping activities and priorities in the earlier phases of a company’s development that situate the company to generate the highest potential profits. As was described with respect to large technology corporations, this incentive may close down possibilities for technology that is designed with health equity in mind. Parthasarathy [57] provided a detailed description of the ways in which a focus on economic gain detracts from the possibility of investing in technologies that meaningfully address the needs of structurally marginalized patients and health care systems. When considered at the scale of investments occurring in digital health around the world, this observation raises serious concerns regarding the likelihood of digital health making meaningful contributions to enhancing equity in access to and outcomes of health care.

### Inequitable Access to High-Speed Internet and Digital Technologies

Where communities lack access to high-speed internet and digital devices, they will be unable to benefit from digital health in many ways [58]. Greene [59] explained that the reasons for a lack of internet access among marginalized populations relate directly to political and economic investments made during the period during which the internet was being established. The internet, relying on a collection of infrastructures, including physical cables, wireless signals, support personnel, and networks of information exchange, was established in 1983, during a time in the United States that led to a commercialized, free market approach to internet access [59]. Although there has been progress in expanding high-speed internet to rural areas and increasing free and affordable access in urban areas [60], a lack of access to high-speed internet and digital devices remains a major barrier to health equity in digital health [58]. As the centrality of high-speed internet access to health and social services becomes more widely understood, scholars have begun to call for high-speed internet access to be formally recognized as a social determinant of health [58].

Digital device availability also contributes to the digital divide. Roughly a quarter of American adults with household incomes below US $30,000 a year (24%) say they do not own a smartphone [61]. If digital health is going to remain a central feature of health care, then establishing sustainable approaches to making devices available to those without access is going to be as important as high-speed internet access [9,17]. A number of charitable programs arose during the COVID-19 pandemic to make digital devices available to those without access [62], but these have largely been temporary responses to the crisis. The question then becomes whether health care funders will fund digital devices for people without access in order to support their engagement with digital health. These 2 related issues of access to high-speed internet and digital devices raise important questions about perceptions of rightful investments by health care funders, health systems, and governments. Our contention is that without investments in these core infrastructures, digital health will exacerbate existing inequities in access to and outcomes of health care over the long term.

### Lack of Investment in Digital Health Literacy

The availability of free or affordable technologies and the internet necessary to use them for health is a crucial starting point for promoting digital health equity. However, the skills and knowledge to use the technology in meaningful ways that promote the attainment of health-related goals are equally important. Digital health literacy has been discussed in different ways, and in this paper, we align with Azzopardi-Muscat and Sørensen [63], who define the concept broadly as “the ability of people to use emerging information and communications technologies to improve or enable health and health care.” In this way, digital health literacy is about possessing the skills and knowledge necessary to navigate digital technologies, digest health-related information, and engage with health care systems in ways that contribute to enhancing one’s health. Even though supporting access to technology for economically marginalized communities has been used in government strategy internationally [59], a lack of coherent investment in digital health literacy aligns with the neoliberal erosion of public services, public education, and coordinated approaches to government [64].

Although governments have progressively built policies on investments in technology as a strategy to promote upward economic mobility [59], digital health literacy initiatives have appeared to arise only sporadically. Government-sponsored initiatives exist internationally, such as the All of Us Research Program in the United States [65] and Health Education England’s collaboration with Libraries Connected [66]. However, the prospect of open-access, well-resourced, and scalable digital health literacy training programs remains elusive internationally. An effort to promote digital health equity at any meaningful scale would require coordinated multistakeholder investments in the delivery of digital health literacy initiatives across communities, health care organizations, and levels of government, and would necessarily include education across proprietary technology offerings.

### Uncertainty About the Future of Equitable Reimbursement for Digital Health Care

In the context of the rapid virtualization of care experienced during the COVID-19 pandemic, the question of the sustainability of multiple digital modalities for ambulatory care delivery was much discussed [67]. In the provincial health care system in Ontario, Canada, physician billing for digital care visits was initially permitted on a temporary basis; that permission was extended multiple times [68]. Importantly, these permissions included the capability of billing for telephone-only digital visits, which has been widely regarded as an equity-promoting policy to ensure those without access to internet-connected digital technologies can retain access to care [67]. However, the government of Ontario announced in March 2022 that telephone-only physician visits would be reimbursed at a substantially lower rate than video visits by provincial health insurance, instituting a major blow to sustained efforts to enable equity of access to digital health services. In other words, where audio-only provision of direct care serves to enhance access for those without the digital devices and high-speed internet access required for video visits, reimbursing audio-only visits at a lower rate will disincentivize providers to continue with audio-only digital care. This policy issue represents a substantial challenge for policy makers, seeking to maintain a high level of quality care while at the same time promoting broad accessibility. Understanding how to address this challenge while maintaining access to telephone-based care options that promote equitable access to services is a central policy issue for the next 10 years [67].

Research in Ontario has shown that the telephone was used appropriately for ambulatory care during the pandemic, and the rise in the use of the telephone was not associated with aberrant or unexpected billing practices among physicians (ie, did not lead to substantial fraudulent billing) [69]. The removal of reimbursement for telephone visits despite this evidence is a symptom of the effort to narrow the scope of government spending, aligning directly with a neoliberal paradigm of public services. However, the question of funding for digital health and digital care is a broader one than reimbursements for telephone visits.

Discussions about funding for telehealth and digital care following the pandemic have emphasized the importance of paying for value, shifting away from volume-based payment toward value-based payment for primary health care [70]. The policy work associated with shifting toward value-based payment as a paradigm for health care systems is immense; as that work proceeds and telephone visits are defunded in the near term, gaps in access to digital care will widen. This reality is compounded by the lack of affordability of internet access and digital devices and low investments in digital health literacy, as discussed earlier. As a future state, a value-based care approach would ostensibly involve health systems or health care providers being paid block payments to manage a patient roster in the way they see fit, using funds to support various modalities of digital health care when such modalities could contribute to enhanced patient outcomes for all [71]. We acknowledge this is an important domain of ongoing research and contend that digital health equity must be a core consideration as value-based care develops in research and policy.

### Justified Mistrust and Exclusionary Design

Beyond the issues of the availability and affordability of digital health technologies is the issue of the documented harms some technologies have caused. Examples linked to health care and health promotion are common, including racism in an automated health care resource allocation algorithm [72] and ineffective sensors on heart rate monitors for people with darker skin [73]. These harms are representations of social systems arising from histories of excluding marginalized communities from commercial activity and quality health care, leading to the development of technologies and services that neglect the needs of such communities or cause them outright harm. Under these circumstances, some communities choose not to engage with digital health technologies as a result of a justified mistrust of their safety and impact [16].

Strategies to address justified mistrust of digital health technologies are well-established and include community engagement and inclusive co-design with affected communities to ensure the cultural sensitivity, accuracy, and use of digital health offerings [16]. However, in the current political economic regime, there is a disincentive to adopt inclusive design strategies across types of commercial actors in digital health (ie, from start-ups to large technology companies). These disincentives arise because inclusive design approaches are costly in that they require additional expertise and time and are challenging to accomplish in meaningful ways [74,75]. As such, barriers to inclusive design remain high. Shifting these incentives and lowering barriers to engagement with inclusive design is a primary challenge in the effort to achieve digital health equity.

## Discussion

### Overview

The challenges to digital health equity arising from a neoliberal political economic regime are multiple and substantial. However, we also find examples across the 6 challenges where efforts are being made within the neoliberal context to prioritize digital health equity by commercial entities, venture capitalists, providers of high-speed internet, digital and health literacy educators, insurers, and inclusive designers. We acknowledge that such examples may be subject to criticism, especially from scholars adopting critical approaches to the digital technology industry. Research in technology ethics, critical data studies, critical digital health studies, and other related fields has emphasized the ways that commercial entities (and especially large technology corporations) use bounded special projects focused on health equity to avoid deeper changes to their business models or the broader health and economic environments [13,18,23,76]. In these instances of “ethics washing” [77,78], or perhaps more accurately, “equity washing,” business models are critiqued for their drive for profits without clear prioritization of health equity, health outcomes, and the quality of health care systems [79].

As scholars anchored by the aim of informing innovations that enhance equity and social justice for people’s health, we are closely attuned to these critiques. However, we have also witnessed the effectiveness of multiple points of intervention in the development of digital health technologies that can orient digital health toward more equitable organization and delivery of care. Furthermore, we are encouraged by the growth in awareness since the onset of the COVID-19 pandemic of the urgent need to promote equitable access to and outcomes of digital health. We are aware of mission-driven digital health initiatives that demonstrate a strong understanding of issues of equitable access, informed consent, and ethical business models. Across our 6 areas, we observe promising opportunities for future work regarding immediate investments in changes to the business models that characterize digital technology under neoliberal capitalism as well as broader changes to health system policies that promote universal access to care.

Large technology corporations like Google are becoming more intentional about their influence on digital health equity. The Google Health Equity team is expanding access to health information through initiatives within Google Search and YouTube and is incorporating insights about the social determinants of health within their Fitbit Health Solutions. Google’s Health Equity Tracker, developed by their health equity team in partnership with the Satcher Health Leadership Institute at the Morehouse School of Medicine [80], 1 of 3 major historically Black medical schools in the United States, provides a real-time dashboard of progress on various health equity indicators. The platform was designed in collaboration with affected communities and is governed by a diverse team of researchers, community members, providers, and technology leaders. The platform presents the opportunity for governments and payers to make strategic investments in enhancing the health of structurally marginalized communities, offering a pathway for digital health technologies to explicitly enhance health equity. Future research could consider how the revenue models that support these collaborations can more equitably yield financial and health benefits for all involved, including the collaborators and individuals sharing data for such initiatives.

Venture capital has explored the potential of social impact investing as a strategy to enhance health equity at local and global levels [81,82]. Social impact investing involves the development of investments in commercial entities that promise to achieve both a financial return on investment and meaningful progress in enhancing social and economic equity [81]. Such investments have included large initiatives in global health [82]. Health systems have also invested in the development of digital health technologies that more comprehensively meet the needs of diverse patients and communities served, such as those developed by accountable care organizations to enhance access to care and reduce hospital admissions [83]. Future research at the nexus of impact investing and digital health should examine the process of venture selection, social, health, and commercial outcomes, and the role of government in directly investing in or developing policies to support equitable digital health venture development [84].

Government, large corporations, and digital health ventures are contributing to digital infrastructure initiatives to improve inequitable access to high-speed internet and digital technologies. For example, the US Affordable Connectivity Program of 2022 recently gave over 48 million households (40% of the country) access to high-speed internet for US $30 per month [85], with a similar government-subsidized program announced in Canada [86]. Certain health plans in the United States allow for free smartphones for Medicaid patients [87], with similar programs occurring only through charitable organizations in Canada [62]. Securing long-term investments and publicizing these offerings to marginalized communities represent crucial investments in digital health equity. Future research should examine factors that promote and inhibit the system-wide scaling of necessary digital infrastructure.

The need for deeper investment in digital health literacy is closely related to the perennial struggle of governments and professional groups to organize systems of health care in ways that support access to care and health promotion for structurally marginalized communities [41]. In our case, this means fractured investments in digital health literacy, applications, and infrastructure that reinforce the existing fragmentation in health systems, with harmful effects magnified for those who are most marginalized [15]. Where initiatives aimed at supporting equitable digital health literacy do exist, they are often temporary, local, and not coordinated across the parties needed to ensure their effective implementation in practice. Digital health literacy may be bolstered not only through health agencies but also through broader educational initiatives. For example, the digital literacy efforts by the US Department of Education’s Literacy Information and Communication System and the Canadian Digital Literacy Exchange program could be expanded to explicitly include digital health literacy. Future research is needed on approaches to the governance of digital health that enable innovation in digital health literacy, with an explicit focus on the needs of structurally marginalized communities.

Questions about inequitable reimbursement for digital health care point to the need for broader changes to health system policies that promote universal access to care. These changes are fundamentally longer-term and deeply intertwined with the distribution of power in the health care and digital technology industries. Given the concentrations of power in these domains, strategies to achieve such high-level change are not immediately clear. Building on the strong critiques provided by critical studies of both the health care and digital technology industries and leveraging insights from diverse fields of work such as institutional change, social movements, and sustainability transitions, urgent work is needed on practical strategies to mobilize change at the industry and policy levels. We suggest that research on these changes in the sphere of digital health has much to learn from interdisciplinary social sciences on the green energy transition, exploring multiple points of intervention to modify business models and social habits that support more sustainable ways of life.

Finally, we see a need for support for the implementation and spread of an inclusive design to address mistrust, and with high urgency. Counteracting our aforementioned disincentives for inclusive design, the US Food and Drug Administration is developing plans to enroll more participants from underrepresented racial and ethnic populations into clinical trials [88]. Design approaches like value-sensitive design are gaining attention from health researchers, such that tensions between stakeholder values can be identified and addressed earlier in design processes [89]. Remaining aware of current critiques and attuned to the need for broader institutional change, this immediate practical work remains focused on collaboration with digital health developers, governments, health systems, and commercial entities to support equity-focused design and culture changes. Future research is needed that acknowledges critiques of co-design in health care while building strong approaches to inclusive design and values-based collaboration with commercial providers of digital health technologies.

### Conclusions

Digital health has become a central feature of health system transformation, presenting an opportunity to steer this transformation in directions that are equity-promoting as a fundamental principle. The role of growth and profit motives and fragmented policy contexts complicate efforts to build coordinated approaches to digital health equity. Nevertheless, the community of researchers, clinicians, community members, and health care leaders committed to health equity holds the power to influence the course of digital health.

As outlined in our paper, there is a substantial body of literature that has drawn on community-engaged and other research approaches to generate strategies to combat digital health inequities at multiple levels. The intellectual work to identify these strategies has already been done and continues to grow. Acknowledging where power wields influence in the broader landscape of digital health, we have proposed future lines of immediate and long-term work aiming to actively promote the advancement of digital health equity. Our recommendations are based on both immediate collaborative work with digital health developers and longer-term efforts to achieve institutional change in the health care and technology industries. Acknowledging the challenges outlined here and investing in the strategies proposed represent important steps toward achieving the goal of digital health equity.
